# Account of Diseases in an Eastern District of London

**Published:** 1802-09-01

**Authors:** 


### ACCOUNT OF DISEASES IN AN EASTERN DISTRICT OF LONDON,

*From July* 20, *to August* 20, 1802.

| | |
|---|---|
| ACUTE DISEASES. | |
| Febris Intermittens Tertiana | 2 |
| Synocha - - - - - - - | 3 |
| Rheumatiſmus Acutus - - | 3 |
| CHRONIC DISEASES. | |
| Tuſſis - - - - - - - - | 6 |
| Dyſpnœa - - - - - - - | 3 |
| Tuſſis cum Dyſpnœa - - | 7 |
| Phthiſis Pulmonalis - - - | 4 |
| Hydrothorax - - - - - | 3 |
| Anaſarca - - - - - - - | 4 |
| Cephalalgia - - - - - | 7 |
| Paralyſis - - - - - - | 3 |
| Epilepſia - - - - - - | 2 |
| Syncope - - - - - - - | 3 |
| Enterodynia - - - - - | 5 |
| Hernia - - - - - - - | 1 |
| Diarrhœa - - - - - - | 3 |
| Hæmorrhois - - - - - - | 2 |
| Dyſuria - - - - - - - | 3 |
| Calculus - - - - - - - | 1 |
| Hernia Humoralis - - - | 1 |
| Impetigo - - - - - - - | 3 |
| Rheumatiſmus Chronicus - | 9 |
| PUERPERAL DISEASES. | |
| Ephemera - - - - - - - | 5 |
| Maſtodynia - - - - - - | 4 |
| Abſceſſus Mammæ - - - | 2 |
| Menorrhagia Lochialis - - | 3 |
| INFANTILE DISEASES. | |
| Angina Trachealis - - - | 1 |
| Pertuſſis - - - - - - - | 3 |
| Rubeola - - - - - - - | 4 |
| Ophthalmia - - - - - - | 5 |
| Tabes Meſenterica - - - | 3 |

The alteration in the ſtate of the weather, and in the temperature of the air, has produced a favourable change in the ſtate of diſeaſes. The various morbid affections of the pulmonary ſyſtem, which have been ſo long protracted by the continuance of a cold and damp atmoſphere, have abated in their number and in their degrees of violence. There are ſtill, however, ſome diſeaſes of the cheſt remaining, which have ſucceeded the meaſles that have very generally prevailed amongſt children.

children. The cafe of Angina Trachealis, mentioned in the lift, terminated fatally. The patient, a fine and healthy boy, after an apparently flight indifpofition of a few days continuance, complained of uneafinefs about his throat, and was foon obferved to breathe with confiderable difficulty. This fymptom was accompanied at firft with a flight degree of fever, and a troublefome cough. Thefe fymptoms continued, and gradually increafed in the degree of aggravation. Upon infpecting the fauces, the tonfils appeared enlarged and inflamed; refpiration became more difficult, and was attended with that peculiar found, which fo ftrongly charaćterifes this difeafe. Leeches were immediately applied to the throat, and a blifter was placed near to the part affećted. Cathartics were repeatedly adminiftered, and a determination to the fkin was kept up by the ufe of antimonials. Confiderable relief was obtained by this plan of treatment. The cough became lefs frequent, refpiration was much relieved, the pulfe was much flower, and the countenance indicated that the general feelings of the patient were more comfortable. Upon an accumulation of vifcid mucus, which impeded refpiration, relief was obtained by gradually increafing the degree of naufea till gentle vomiting was excited. But though relief was repeatedly obtained, and appearances were favourable for a fhort time, yet, as is too commonly the cafe in this difeafe, thefe proved fallacious, and were foon fucceeded by an aggravation of fymptoms which terminated in death.

Having obtained permiffion to infpect the difeafed part, a confiderable portion of the trachea was removed, which, upon being opened, difcovered marks of inflammation throughout its furface. Upon the fuperior part fome purulent matter appeared, underneath which, and extending fome way down the trachea, a thick membranous fubftance was difcovered adhering very firmly, fo as with difficulty to be removed by the probe.

